# Sirolimus and Everolimus Pathway: Reviewing Candidate Genes Influencing Their Intracellular Effects

**DOI:** 10.3390/ijms17050735

**Published:** 2016-05-14

**Authors:** Simona Granata, Alessandra Dalla Gassa, Amedeo Carraro, Matteo Brunelli, Giovanni Stallone, Antonio Lupo, Gianluigi Zaza

**Affiliations:** 1 Renal Unit, Department of Medicine, University/Hospital of Verona, 37126 Verona, Italy; simona.granata@univr.it (S.G.); alessandra.dallagassa@univr.it (A.D.G.); antonio.lupo@univr.it (A.L.); 2 Liver Transplant Unit, Department of General Surgery and Odontoiatrics, University/Hospital of Verona, 37126 Verona, Italy; amedeo.carraro@ospedaleuniverona.it; 3 Department of Pathology and Diagnostics, University of Verona, Azienda Ospedaliera Universitaria Integrata, 37126 Verona, Italy; matteo.brunelli@ospedaleuniverona.it; 4 Nephrology, Dialysis and Transplantation Unit, University of Foggia, 71122 Foggia, Italy; giovanni.stallone@unifg.it

**Keywords:** mTOR inhibitors, sirolimus, everolimus, transplantation, genes

## Abstract

Sirolimus (SRL) and everolimus (EVR) are mammalian targets of rapamycin inhibitors (mTOR-I) largely employed in renal transplantation and oncology as immunosuppressive/antiproliferative agents. SRL was the first mTOR-I produced by the bacterium *Streptomyces hygroscopicus* and approved for several medical purposes. EVR, derived from SRL, contains a 2-hydroxy-ethyl chain in the 40th position that makes the drug more hydrophilic than SRL and increases oral bioavailability. Their main mechanism of action is the inhibition of the mTOR complex 1 and the regulation of factors involved in a several crucial cellular functions including: protein synthesis, regulation of angiogenesis, lipid biosynthesis, mitochondrial biogenesis and function, cell cycle, and autophagy. Most of the proteins/enzymes belonging to the aforementioned biological processes are encoded by numerous and tightly regulated genes. However, at the moment, the polygenic influence on SRL/EVR cellular effects is still not completely defined, and its comprehension represents a key challenge for researchers. Therefore, to obtain a complete picture of the cellular network connected to SRL/EVR, we decided to review major evidences available in the literature regarding the genetic influence on mTOR-I biology/pharmacology and to build, for the first time, a useful and specific “SRL/EVR genes-focused pathway”, possibly employable as a starting point for future in-depth research projects.

## 1. m-TOR Inhibitors (mTOR-I): Clinical Aspects

Sirolimus (SRL) and everolimus (EVR) are drugs frequently employed in renal transplantation that inhibit the mammalian target of rapamycin (mTOR), a serine-threonine kinase implicated in cell growth, protein synthesis, proliferation, and apoptosis.

Sirolimus (SRL) was the first mammalian target of rapamycin inhibitor (mTOR-I) produced by the bacterium *Streptomyces hygroscopicus* and approved for renal transplantation. Everolimus (EVR), derived from sirolimus, contains a 2-hydroxy-ethyl chain in the 40th position that makes the drug more hydrophilic than SRL and increases oral bioavailability by approximately 10%–16% [1].

Both bind to FK506-binding protein 12 (FKBP12, encoded by the *FKBP1A* gene), and the SRL/FKBP12 and EVR/FKBP12 complexes each bind directly to mTOR, blocking cell cycle progression from G1 to the S phase and cellular proliferation [2,3].

The introduction of these pharmacological agents in solid organ transplantation had a positive impact on renal function, mainly determined by a reduced employment of nephrotoxic calcineurin inhibitors (CNIs) [4,5,6].

In patients with chronic allograft dysfunction (CAD), a condition characterized by a functional and anatomical deterioration of the graft occurring at least 3–6 months post-transplant, CNI withdrawal and mTOR-I conversion caused better graft survival and reduced chronic histological alterations [7,8]. Additionally, the intra-graft α-smooth muscle actin (α-SMA) expression was downregulated after the switch to SRL, suggesting a favorable effect in preventing the development of renal fibrosis [9].

Moreover, the employment of mTOR-I has considerably decreased the rate of viral infections (e.g., cytomegalovirus and BK virus) [10,11,12,13] and cardiovascular complications (e.g., hypertension and left ventricular hyperplasia) [14,15,16,17] in solid organ transplant recipients.

Furthermore, because of the aberrant hyper-activation of mTOR signaling in various types of cancers, a specific inhibition by mTOR-I could represent a valuable treatment for these pathologies. The anti-neoplastic efficacy is also related to the inhibition of angiogenesis through the downregulation of VEGF release together with reduced endothelial sensitivity to this factor [18].

Clinical trials are ongoing with SRL and EVR (together with temsirolimus and deforolimus) in different kinds of tumors. EVR and temsirolimus have received FDA approval for the treatment of patients affected by renal cell carcinoma [19,20]. EVR has also been approved for several neurological/neuroendocrine tumors.

A second generation of mTOR-I able to simultaneously inhibit mTORC1 and mTORC2 [21,22] are currently in clinical trials demonstrating encouraging anti-cancer potentials.

Although, experimental procedures employing mTOR-I have clearly demonstrated that the modulation of the PI3K/Akt/mTOR pathway could be a good target of anticancer therapy, the clinical responsive rates to these medications have been poor and highly variable in several tumors.

As well, the anticancer efficacy of mTOR-I seems to be limited to their cytostatic and weak cytotoxic activities, so the clinical effect is stabilization rather than regression. This makes them particularly useful for the immunosuppressive treatment of patients developing malignancies after organ transplantation [23]. In the 2013 Australian and New Zealand Data report [24], cancer represented between 33% and 35% of all deaths beyond the first year of transplant. In an analysis combining different US registry data [25], the overall cancer risk among solid organ transplant recipients was 2.1 times higher when compared to the general population.

In the Rapamune Maintenance Regimen trial, early cyclosporin A withdrawal (3 months post-TR) followed by the introduction of SRL caused fewer malignancies compared with a combined SRL plus cyclosporin A immunosuppressive schema [26].

Additionally, Campistol *et al.* [27] reported less incidence of cancer after long-term follow-up (5 years) in SRL-treated patients.

Similar results were also found following late conversion from CNI to mTOR-I in the CONVERT trial [28].

## 2. The Biological Effects of mTOR-I

The discovery of mTOR and the understanding of its biological functions have been facilitated by the use of SRL and EVR (and other analogs) in organ transplantation and oncology.

As largely reported by several basic science and translational research studies, mTOR constitutes the catalytic core of two multiproteins complexes, mTOR complex 1 (mTORC1) and 2 (mTORC2), which have different targets and sensitivity to rapamycin.

mTORC1 includes RAPTOR [29,30], MLST8 [31], PRAS40 [32], and DEPTOR [33]. The pivotal upstream regulator of mTORC1 is TSC1 (hamartin) and TSC2 (tuberin) with its downstream target Rheb GTPase. When Rheb is bound to GTP, mTOR kinase activity is stimulated. TSC1/TSC2 converts Rheb into its inactive state, inhibiting mTORC1 [34,35]. Many factors (e.g., nutrients and growth factors) activate mTORC1 [36] through the PI3K-PDK1-AKT pathway with the inactivation of the TSC1/TSC2 complex. mTORC1 senses nutrient signals through the RAS-related GTP-binding protein (RAG) family and translocates to the surface of the lysosome, and it is activated by RHEB [34,37]. A high cellular energetic state (high ratio of ATP to AMP), hindering the activation of AMPK, activates mTORC1 [37].

mTORC2 includes the RICTOR [38], MAPKAP1 [39], PRR5/PRR5L [40], Mlst8, and Deptor. This complex is less sensitive to acute treatment with rapamycin and its analogues, while chronic rapamycin treatment inhibits mTORC2 function by acting on complex integrity [41,42]. mTORC2 signaling, mainly activated by growth factors, controls several processes including cellular survival, proliferation, and organization of cytoskeleton [43]. mTORC2 directly phosphorylates Akt(S473), modulating cell survival, apoptosis, growth, and proliferation [44,45]. mTORC2 regulates the organization of the actin cytoskeleton through phosphorylation of PKCα [46,47], and it is a regulator of neutrophil polarity and chemotaxis through cAMP/RhoA-signaling [48]. mTORC2 also directly activates serum and SGK1, a kinase implicated in the control of ion transport and cellular proliferation [49].

mTORC2 is required for epithelial to mesenchymal transition (EMT) in response to TGF-β, which induces mTORC2 kinase activity to mediate phosphorylation of Akt(S473) through PI3K [50,51,52,53].

While inhibition of mTORC1 is universal, mTORC2 inhibition is tissue-specific [41]. It has been recently proposed that this difference could be due to different levels in the expression of FKBPs. In particular, cells responsive to mTORC2 inhibition have a higher FKBP12-FKBP51 ratio compared to cells insensitive to mTORC2 inhibition by rapamycin [54].

**Figure 1:**
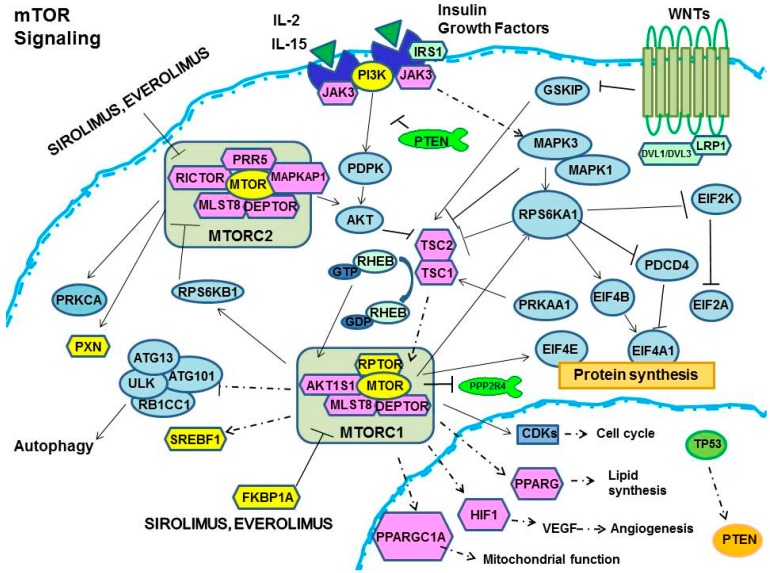
Intracellular pathway reporting major candidate genes interacting with sirolimus (SRL) and everolimus (EVR). Solid lines indicate direct interactions, whereas dashed lines show indirect effects.

The FKBP12-EVR or FKBP12-SRL complex allosterically inhibits mTORC1 activity and signaling by weakening the interaction between mTORC1 and RAPTOR [30,55]. This interaction inhibits downstream functions and pathways including: 1. protein synthesis; 2. HIF-1 and VEGF (*VEGFA*)*-dependent* regulation of angiogenesis; 3. lipid biosynthesis; 4. mitochondrial biogenesis and function; 5. cell cycle and growth; and 6. autophagy. All of these effects are modulated/regulated by a large genetic encoding network (Figure 1) (Table 1) [56,57,58,59,60,61,62,63,64,65,66,67,68,69,70,71,72,73,74,75,76,77,78,79,80,81,82,83,84,85,86,87,88,89,90,91,92,93,94,95,96,97,98,99,100,101,102,103,104,105,106,107,108,109,110,111,112,113,114,115].

### 2.1. Control of Protein Synthesis by m-TOR-I

Although protein synthesis is a well-known process regulated by mTOR pathway, the exact biological machinery involved in protein-system modulation by m-TOR-I is not completely defined. It is unquestionable that mTOR-I may act by regulating different sites of phosphorylation in the protein biosynthetic cellular process.

Protein synthesis is conventionally divided into three main stages: (1) initiation; (2) elongation; and (3) termination. The limiting step is the translation initiation when the ribosome is recruited to the mRNA [116,117]. This process needs the assembly of the eukaryotic translation initiation factor 4F (eIF4F) on 5′ mRNA. This complex includes: eIF4E (*EIF4E*), eIF4G (*EIF4G1*), and eIF4A (*EIF4A1/2*). The inhibitory 4E binding protein 1 (4EBP1 or eIF4EBP1), the target of mTORC1, binds to eIF4E and interferes with the interaction between eIF4E and eIF4G. The phosphorylation of 4EBP1 at specific sites (Ser^65^/Thr^70^) by mTORC1 causes its separation from eIF4E, leaving eIF4G and eIF4A for recruitment [118].

Other downstream targets of mTORC1, directly involved in protein synthesis, are ribosomal protein S6 kinases S6K1 (*RPS6KB1*) and S6K2 (*RPS6KB2*) [119]. mTORC1 phosphorylates and activates S6Ks [120] that in turn phosphorylate several proteins linked to mRNA translation including ribosomal protein S6 (*RPS6*), eIF4B, [121], eEF2K, and programmed cell death 4 (Pdcd4; *PDCD4*). S6K1 and RPS6 are not required for translation regulation [122], but it has been reported that they regulate cell size and proliferation since cells isolated from RPS6P^−/−^ displayed defective cell growth [123]. S6K1 regulates translation initiation by phosphorylating the cap binding complex component eIF4B at S422, promoting the recruitment of eIF4B to eIF4A at the translation initiation complex where it functions as a cofactor of eIF4A and increases its helicase activity [124]. S6K1 phosphorylates and inactivates eEF2K, which negatively regulates eukaryotic elongation factor 2 (eEF2) and thus regulates the elongation step of translation [125]. PDCD4 binds to eIF4A and inhibits its helicase activity [126]. This results in PDCD4 ubiquitination and degradation mediated by the E3 ubiquitin ligase β-TrCP [127].

Another target of S6K1 is SKAR (*POLDIP3*), a biological factor that recruits activated S6K1 to new synthetized mRNAs [128,129].

Additionally, although it has been extensively reported that mTORC1 directly phosphorylates 4EBP1, it has been shown that this protein has different sites of phosphorylation, some of which are insensitive to rapamycin [130].

It is noteworthy that the use of compounds able to inhibit the protein kinase activity of mTOR are much more effective in protein synthesis inhibition compared to rapamycin. This may be explained by the fact that rapamycin could be able to interfere with signaling from mTOR to 4EBP1 instead of a direct phosphorylation by mTOR [131,132].

The control of translation and protein synthesis, in particular through 4EBP1 phosphorylation is one of the possible mechanisms that play a role in mTOR-I-dependent regulation of angiogenesis.

### 2.2. mTOR-I Regulation of Angiogenesis

Angiogenesis is the neo-synthesis of endothelial cells and new blood vessels that occurs in physiological conditions (e.g., growth/development and wound healing) and, unfortunately, in several pathological states including tumors genesis and metastasisation. Additionally, this event plays a protective role against ischemic injury.

Several factors are involved in this process, but HIF-1 and VEGF play a central role. Both factors are regulated by the mTOR pathway, and they could be indirectly modulated by agents acting at this cellular level.

HIF-1 is a dimeric protein complex consisting of HIF-1α and HIF-1β subunits and acts as a regulator of several genes involved in maintaining homeostasis following changes of oxygen concentration [133]. Under normoxic conditions, the α-subunit interacts with the E3 ubiquitin ligase complex through its oxygen-dependent degradation (ODD) domain and undergoes degradation via the ubiquitin-proteasome pathway [134]. Contrarily, HIF-1β is a constitutively expressed nuclear protein. HIF-1 also plays a role in immune-response, vascularization, and anaerobic metabolism [135,136].

The oxygen-dependent turnover of HIF-1α is controlled by physiological conditions and regulators including mTOR [137]. In fact, as recently reported, the mTOR pathway mediates the cellular adaptation to oxygen- and nutrient-poor environmental conditions [138,139,140].

HIF regulates the transcription of dozens of target genes including VEGF [141], an essential element for early vascular development [142,143].

Due to the central role of VEGF in solid tumors, several anti-VEGF therapies have become part of anticancer regimens, including EVR [144,145]. mTOR-I, reducing the expression of VEGF [146,147] through HIF-1α, could reduce cellular adaptation to hypoxia with a consequent remarkable effect on tumor growth, invasiveness, and cancer metastasisation. However, the exact mechanisms by which mTOR regulates HIF are elusive. mTORC1 drives HIF-1α transcription via phosphorylation of STAT3 on Ser^727^ during hypoxia [148,149].

Moreover, it has been suggested that mTOR had no effect on HIF-1α stability and promotes the accumulation of HIF-1α protein, mainly by enhancing its synthesis [149,150]. In particular, Düvel *et al.* showed that the increased translation of HIF-1α was mostly due to the increment of 4EBP1 phosphorylation and cap-dependent translation, dependent on mTORC1 [151].

Additionally, S6K1 is an important mediator of HIF-1α translation, but inhibition of S6K1 has no effect on VEGF levels, suggesting that VEGF expression is mediated via both HIF-1α-dependent and -independent mechanisms [148,152,153].

### 2.3. mTOR Inhibition and Lipid Biosynthesis

Several studies have tried to define the biological bases of the mTOR regulation of lipid biosynthesis and the effects of mTOR inhibition.

mTORC1 activates SREBP-1, a transcription factor that regulates the expression of genes required for cholesterol, fatty acid, triglyceride, and phospholipid synthesis and peroxisome proliferator-activated receptor-γ (PPAR-γ; *PPARG*)-activating ligands [154,155,156].

The exact mechanism underlying mTORC1 regulation of SREBPs still remains to be determined. S6K1 plays a crucial role in this process since S6K1^−/−^ mice fed with a high fat diet did not gain weight. This seemed to be due to an impaired generation of adipocytes [157,158,159]. Another hypothesis is that mTORC1 could promote SREBP1 processing through the induction of endoplasmic reticulum (ER) stress mainly triggered by an elevated protein synthesis. ER stress promotes SREBP1 activation in the liver, inducing upregulation of lipogenic genes [160,161]. Additionally, mTORC1 regulates SREBPs through lipin-1 (*LIPIN1*), a phosphatidic acid phosphatase that promotes triglyceride synthesis and acts as a transcriptional coactivator for many transcription factors, including PPAR-γ [162].

In adipocytes, lipin-1 is activated by a great number of stimuli through the mTOR pathway [163]. When active, mTORC1 phosphorylates lipin-1 with its consequent nuclear exclusion and activation of SREBP-dependent gene transcription [164,165].

Recent reports have also suggested the involvement of mTORC2 in the control of lipid biosynthesis through AKT. Although the exact mechanisms involved are not yet clarified, several observations have been well accepted. AKT decreases the expression of *Insig2a*, facilitating the processing of SREBP1 [166,167]. AKT also phosphorylates SREBP1, which promotes SREBP1 transport from the ER to the Golgi [168]. Finally, AKT inhibits proteasomal degradation of SREBP1 mediated by glycogen synthase kinase 3 [169]. Rapamycin stops the AKT-related nuclear localization of SREBP1, the expression of genes of the lipogenesis pathway, and the production of many lipids [170]. mTORC1 signaling is a critical step in adipocyte differentiation at least in part through PPAR-γ [171,172,173,174,175,176,177,178].

Despite these effects, it has been reported that, in transplantation, the dyslipidemia induced by mTOR-I could increase cardiovascular diseases [179]. These drugs increase LDL, cholesterol, and triglycerides in approximately 40%–75% of patients who receive this therapy [180,181,182]. The pathogenesis of dyslipidemia is unclear, but an upregulation of circulating levels of apolipoprotein B-100, apolipoprotein C-III (an inhibitor of lipoprotein lipase), and adipocyte fatty acid-binding protein 2 have been reported [183,184].

Interestingly, mTOR inhibition downregulated lipoprotein lipase in adipose tissue with a consequent impairment in the ability to hydrolize, take up, and store circulating lipids [185].

It is noteworthy that hyperlipidemia is reversible and dose-dependent. A study by Morrisett *et al.* observed that cholesterol and triglyceride levels increase after 2–4 weeks of initiation of therapy, and this alteration reverted to near-baseline levels within 8 weeks after discontinuation of treatment [184].

Although not completely clarified, it is unquestionable that mTOR signaling is a pivotal player in the control of cellular energy homeostasis; therefore, in the last several years, researchers have also focused on the interaction between mTOR and the mitochondria, often referred to as the “powerhouse” of the cell.

### 2.4. Biochemical mTOR-I-Related Mitochondrial Biogenesis and Functional Regulation

Mitochondria are organelles involved in numerous functions: ATP synthesis by oxidative phosphorylation, fatty acids β-oxidation, synthesis of heme, apoptosis, synthesis of steroid hormones, nitrogen balance through urea cycle, and Ca homeostasis.

Mitochondrial biogenesis and activity are regulated by several transcription factors (including NRFs, ERRs, YY1) [186] coordinated by the transcriptional coactivators PGC1-α (*PPARGC1A*), PGC1-β (*PPARGC1B*) and PRC (*PPRC1*), which have many functions including chromatin modification by posttranslational histone acetylation, RNA polymerase II complex interaction, mRNA processing, and the recruitment of other transcriptional coactivators [187].

mTOR inhibition affects translation of several but not all mitochondrial genes. In particular, only the inhibition of RAPTOR (and then mTORC1) suppressed the translation of various nuclear-encoded mitochondrial regulators such as TFAM, mitochondrial ribosomal proteins, and components of the complex I and V [188]. Likewise, functional assays revealed that mTORC1 inhibition decreased ATP levels, mtDNA content, and both coupled and uncoupled respiration, whereas RICTOR depletion had no effect [188,189].

Several mechanisms have been proposed underlying this effect, not necessary mutually exclusive. mTORC1 stimulates mitochondrial biogenesis and activity through a direct interaction with YY1 and PGC1-α. mTOR inhibition by rapamycin was reported to prevent this physical interaction, resulting in a reduced expression of mitochondrial genes [190]. It has been reported that mTOR co-localizes with mitochondria and is sensitive to mitochondrial dysfunction. This localization permits the mTOR activity to be modulated by the redox status of the cell [191]. Recently, it has been demonstrated that mTOR could mediate the activity of mitochondria through the phosphorylation and consequent activation of the anti-apoptotic protein Bcl-xl (*BCL2L1*) [192].

### 2.5. m-TOR-Is Control Cell Cycle and Growth

Pharmacological inhibition of mTOR induces cell-cycle G_1_-arrest in lymphocytes, while in most other cells it induces only a delay of the cell cycle progression [2,3].

Inhibition of mTORC1 and PI3K, independently, reduced cell size and led to an accumulation of cells in G_1_ phase. Fingar *et al.* proposed that mTOR could represent the central coordinator between cell cycle and growth. Since S6K1 and 4EBP1/eIF4E pathways control translation, the increment of expression of protein regulators of the cell cycle could be a mechanism by which cell cycle progression is coupled to cell growth [193,194]. In addition, these factors regulate mRNA synthesis and processing: eIF4E regulates nucleocytoplasmic transport of mRNA transcripts. S6K1 also interacts with proteins that couple transcription, splicing, and RNA export [119,194].

More recently, another proposed mechanism by which mTOR regulates cell proliferation involved mTORC2 and FOXO3a. This transcription factor belongs to the Fork head box O (FoxO) family which consists of FoxO1 (*FOXO1*), 3 (*FOXO3*), 4 (*FOXO4*), and 6 (*FOXO6*) [195]. In particular, FoxO3a stimulates the gene expression of cyclin-dependent kinase inhibitors (CDKIs), thereby blocking the cell cycle progression. mTORC2 activates AKT and SGK1 that phosphorylate FoxO3a at Thr^32^/Ser^253^ and Ser^314^, respectively [196,197]. These modifications determine a nuclear export of FoxO3a with the consequent inhibition of CDKI.

Additionally, when mTORC2 is activated, RICTOR mediates the ubiquitination and degradation of SGK1 [198], causing a diminution of FoxO3a phosphorylation at Ser^314^. Consequently, this transcription factor is retained in the nucleus and may activate the expression of CDKI [199].

### 2.6. mTOR-I and Autophagy

Autophagy is a cellular digestion process finalized to remove damaged macromolecules and organelles followed by a recycle of cellular components. This complex process provides energy and molecular building blocks during nutrient starvation and other stress conditions [200,201].

This cellular process is divided in three main classes: macroautophagy, microautophagy, and chaperone-mediated autophagy. The best-studied class is macroautophagy.

During the first step of macroautophagy, the autophagosome, characterized by the double-membrane vesicle including soluble materials or organelles, is built. Subsequently, this structure is fused to the lysosome to become an autolysosome. At this stage, all material included is degraded [202].

In mammals, the initial step in the autophagosome formation is the assembly of ULK complex containing ULK, FIP200, ATG13, and ATG101.

mTORC1 interacts directly with ULK complex under nutrient-enriched conditions and phosphorylates ULK1 and ATG13, thereby inhibiting ULK1 function [203,204]. The proposed mechanism could be a local perturbation of the protein–protein interaction that interferes with ULK recognition of FIP200 [205]. Therefore, the inhibition of mTORC1 enhances the kinase activity of ULK1/2 and triggers the phosphorylation of Atg13 and FIP200 and autophosphorylation of ULK [203,204,205].

In addition, mTORC1 phosphorylates autophagy/beclin 1 regulator 1 (AMBRA; *AMBRA1*), an important regulator of autophagy mechanism, maintaining it in an inactive state [206].

mTORC1 also regulates autophagy at the transcriptional level by modulating the localization of TFEB, a regulator of lysosomal and autophagy protein gene expression [207,208]. Its activity is regulated by nutritional status of the cell, and phosphorylation regulates the shuttling cytoplasm to the nucleus [209]. mTORC1 phosphorylates TFEB at Ser^142^ and Ser^211^, resulting in cytoplasmic sequestration of the transcription factor [210].

Interestingly it has been reported that the termination of autophagy could be mTOR-mediated because the release of the constituents of macromolecules degraded by autophagosomes can in turn reactivate mTORC1, which terminates autophagy [211].

Cao *et al.* have reported that EVR enhances the cytotoxic effects of radiation on tumor cells (PC-3 and DU145) probably through a drug-related induction of autophagy [212].

## 3. MicroRNA (miRNAs) and mTOR

miRNAs are small (of approximately 22 nucleotides) non-coding RNAs that regulate gene expression at the post-transcriptional level and recognize mRNA targets by binding with partial complementarity to the 3′UTR of the target gene. This leads to an inhibition of translation and facilitation of degradation of the target mRNA [213,214]. Therefore, based on the aforementioned characteristics, it is not surprising that miRNAs regulate several drug functional genes. The study of the influence of miRNAs in drug efficacy has built the basis for a new discipline called “miRNA pharmacogenomics” [215].

Numerous studies indicate that mTOR and its signaling pathway are regulated by miRNAs.

Totary-Jain *et al.* have recently demonstrated that rapamycin resistance, developed by long-term rapamycin treatment, is associated with an extensive reprogramming of the miRNA transcriptome, with the hyper-expression of miR-17-92 and related clusters and down-expression of tumor suppressor miRNAs such as miR-143, miR-29, and miR-22 [216,217].

Recently, Zou *et al.* identified eight miRNAs that were specifically modulated by mTORC2, but not mTORC1. In particular, miR-9-3p reduces the expression of E2F1. Therefore, they suggest that mTORC2 inhibition or depletion stimulates the expression of miR-9-3p, which directly targets E2F1 to promote genotoxic drug-induced apoptosis [218].

Moreover, rapamycin reduces cell viability and proliferation in endothelial cells through the upregulation of miR-21 [219,220]. Likewise, EVR induces apoptosis directly, regulating the expression levels of apoptosis-related microRNAs such as miR-145 and miR-15a in renal cancer cells [221]. Rapamycin impairs the muscle regeneration through the downregulation of the transcription of miR-1 [222].

In addition, several miRNAs target different components of the mTOR pathway. miR-7 inhibits tumorigenesis and cancer metastasis in hepatocellular carcinoma by blocking PIK3CD, mTOR, and p70S6K [223]. miR-99a was downregulated in both oral squamous cell and renal cell carcinoma, and its low expression was associated with poor outcomes in patients with renal tumor. The restoration of miR-99a has antitumor properties through the inhibition of the mTOR pathway [224,225]. In certain tumors mediated by the upregulation of the tyrosine kinase c-Src, it has been suggested that under-expression of miR-99a could be determined by the activation of Src-related pathways with the consequent upregulation of mTOR and the activation of protein synthesis and tumor growth [226]. miR-7a is a major form of mature miR-7 expressed in adult pancreatic islets, targeting the mTOR signaling pathway and negatively regulating adult β-cell proliferation. This effect was reversed by rapamycin [227]. In colorectal cancer, miR-144 downregulation is associated with poor prognosis, probably through the activation of mTORC2 [228]. In oral squamous cell carcinoma, the epigenetic silencing of tumor suppressor miR-218 is likely to be an important mechanism of carcinogenesis and cancer progression at least partly involving the activation of mTORC2-Akt signaling [229].

The antitumor miR-100 represses mTOR signaling in endothelial and vascular smooth muscle cells, showing antiangiogenic function [230]. This miRNA is downregulated in clear-cell ovarian carcinoma cell lines, and it has been reported that its overexpression represses mTOR mRNA and protein levels, enhancing the sensitivity to EVR [231].

In esophageal squamous cell carcinoma (ESCC), miR-99a and miR-100 are downregulated and correlated with poor prognosis. These miRNA suppress the expression of mTOR in a post-transcriptional manner and induce apoptosis, thereby decreasing the proliferation of ESCC cell lines, and may play an important role in suppressing the tumor growth [232].

## 4. Pharmacogenetics/Genomics and mTOR-I

Pharmacogenetics involving mTOR inhibitors have primarily focused on the effects of SNPs in *CYP3A4*, *CYP3A5*, and *ABCB1* genes on the metabolism and pharmacokinetic of these medications [233,234,235,236,237].

In particular, it has been reported that the post-treatment SRL concentration-dose ratio was influenced by the *CYP3A4* genotype and resulted higher in patients carrying the wild-type genotype (CYP3A4*1/*1) compared to those with CYP3A4*1B mutant alleles. This difference is probably due to a higher enzymatic activity in subject-carrying mutant alleles [238].

The *CYP3A5* gene contains a SNP in intron 3 (CYP3A5*3) that affects RNA splicing with a consequent synthesis of an enzyme with reduced activity [239]. Patients carrying CYP3A5*1 showed a lower SRL concentration-dose ratio compared to CYP3A5*3/*3 carriers, suggesting that they require a lower SRL daily dose to reach sufficient blood concentration [236,237]. Interestingly, this genotype has no influence on EVR metabolism and pharmacokinetics [240,241], and several studies did not find any influence of genetic polymorphism on SRL pharmacokinetic in patients also treated with CNI [235,237].

Furthermore, Sam *et al.* [242] stated that patients carrying at least one ABCB1 3435T allele have a higher mean SRL concentration-dose ratio compared to patients with the 3435CC genotype and in IL-10-1082GG homozygotes compared with -1082A heterozygotes and homozygotes.

Authors suggest that this effect was due to an augmented IL-10 expression with consequent reduced CYP3A activity and SRL metabolism in patients with this genotype [243,244]. However, other studies have found no association of the *ABCB1* 3435C>T SNP with a SRL concentration-dose ratio [238,245].

No significant studies have been published regarding the polygenic influence on pharmacodynamics.

In the last 10–15 years, nephrology researchers have utilized genomics and transcriptomics methodologies to discover new therapeutic targets for immunosuppression and to identify tools to achieve the so-called “personalized medicine”.

Nevertheless, from the current literature, we are still not ready for a clinical employment of these “omics” technologies or to individualize mTOR-I treatment based on them. We believe that, in the next few years, national and international clinical studies or trials should be undertaken to translate results of pharmacogenetics/genomics studies in clinical practice.

## Figures and Tables

**Table 1:** Genes involved in the intracellular pathways modulated by everolimus (EVR) and sirolimus (SRL).

Gene Symbol	Genetic Variants	Ref.	Clinical Impact
*AKT1*	G49A	[56]	Association with primary breast tumor
	rs2498804	[57]	Association with survival and response to therapy in Squamous cell carcinoma of the head and neck
	rs2498804	[58]	Association with reduced anti-apoptotic efficiency and higher risk of disease reactivation after natalizumab discontinuation in multiple sclerosis patients
	rs2498786	[59]	Association with Alzheimer′s disease risk
	rs1130214, rs3803300; rs3730358	[60]	Association with risk of oral squamous cell carcinoma and survival
	rs2498804	[61]	Association with the risk of recurrence and survival in gastric cancer patients
	copy number gains of the AKT1 locus at 14q32.33	[62]	Association with elevated mRNA expression of *AKT1* in intracranial germ cell tumours
	rs2498804; rs2494732	[63]	Association with risk of brain metastasis in non-small cell lung cancer
	A968G; G49A	[64]	SNPs found in Müllerian adenosarcoma
	rs1130214	[65]	This SNP influences metabolic variables and their responses to aerobic exercise training in older, previously sedentary individuals
	rs3803304	[66]	Association with lifespan
	rs3803304; rs2498804; rs1130214	[67]	Association with recurrence risk, survival and response to chemoradiotherapy in esophageal cancer patients
	rs2498801	[68]	Association with increased risk of endometrial cancer
	rs3730358; rs2498799	[69]	Association with resistance to apoptosis contributing to the low response of caucasian EBV-transformed B lymphocyte cell lines to radiation therapy
	rs3730358	[70]	Association with early age of cancer (breast and/or ovarian) onset in *BRCA1/2* carriers
	rs3730358	[71]	Association with lung cancer risk
	rs2494738	[72]	AKT1 rs2494738 (G>A) & PDK1 rs11904366 (G>T) in combination with dietary fat and carbohydrate, influence the risk of both colon and rectal cancer
*AKT2*	rs892119	[67,68]	Association with high recurrence risk and negative survival rate in esophageal cancer patients and in endometrial cancer
	rs3730050	[73]	Association with overall survival in metastatic bladder cancer patients
	rs8100018, rs3730051	[74]	Association with polycystic ovary syndrome
*AKT3*	rs2994329	[75]	Association with bladder cancer risk
	rs2125230	[76]	Synergistic interaction between *AKT3 rs2125230-PRKCQ rs571715* and prostate cancer aggressiveness
	rs4132509; rs3766673; rs12031994; rs4430311; rs1058304; rs2345994	[77]	Association with increased risk of renal cell carcinoma
*FKBP5*	rs352428	[78]	Association with a decreased transcriptional activity and low FKBP5 expression resulting in poor response to serotonin reuptake inhibitors in patients with major depressive disorder
*MTOR*	G6981T; T4358C; A6139T; A5941G; T6643C	[62]	Mutations found in intracranial germ cell tumours
	rs11121704; rs2295080	[67]	Association with poor survival and poor response to taxane in esophageal cancer patients
	AGAAA haplotype (rs1770345/rs2300095/rs2076655/rs1883965/rs12732063)	[79]	This haplotype, in addition to SRL trough levels, was significantly associated with a decrease in haemoglobin levels in renal transplant recipients switched from a calcineurin inhibitor to sirolimus
	rs2295080	[80,81]	Association with gastric cancer risk and renal cell carcinoma susceptibility by modulating the endogenous MTOR expression level
	rs1883965	[82]	Association with an increased risk of gastric cancer
	rs2024627; rs1057079	[83]	Association with colon cancer
*PI3KCA*	A3140G; G1633A; G1624A, A3140T; T1035A; A1637C; G1633C	[56]	Association with primary breast tumor
	rs7621329	[61]	Association with the risk of recurrence in gastric cancer patients
	rs2699887	[63]	Association with risk of brain metastasis in non-small cell lung cancer
	rs6443624; rs9838411; rs2699887	[68]	Association with risk, survival and recurrence of endometrial cancer
	rs6443624	[84]	Association with survival in renal cell carcinoma patients treated with everolimus
	G1624A; G1633A; A1637C; A3140G; A3062G; G3145C; A3140G	[85]	These mutations are very common in breast cancer and associated with estrogen receptor(+) status, small size and the risk to relapse
	del325-327; gene amplification; A1634G; G1633A; A1634C; A3140T; A3140G; T335A; G1638T; A3062C; C3074A; T3107C; A3140G	[86]	A1634G; G1633A; A1634C; A3140T; A3140G are mutations found in Colorectal cancer. T335A; G1638T; A3062C; C3074A; T3107C; A3140G; T3141G are mutations found in endometrial carcinomas
	amplification of the 3q26 region, increased PIK3CA copy number	[87]	Association with high pStathmin(S38) level, a marker of poor prognosis in endometrial cancer patients
	C112T; G113A; G263A; C311G; G317T; G323C; del332-334; G353A; G365A; C370A; G1048C; T1132C; T1258C; G1357C; C1616G; G1624A; A1625G; A1625T; G1633A; A1634G; G1635T; C1636A; A1637C; C1981A; A2102C; G2702T; T3022C; A3073G; C3074A; G3129T; C3139T; A3140G; A3140T; G3145A	[88]	These mutations have been found in several human cancers
	A3140G; G1624A; C1636A; G1633A; G3145A; G1645A; G3129C	[89]	These mutations are highly frequent in patients with endometrial, ovarian, colorectal, breast, cervical cancer, NSCLC, and squamous cell cancer of head and neck. The response rate was significantly higher for patients with PIK3CA mutations treated with PI3K/AKT/mTOR pathway inhibitors
	G1624A; G1633A; A3140G	[90]	Association with worse time to progression in the HER2-positive patients with metastatic breast cancer treated with Trastuzumab
*PI3KCA*	rs4855094, rs7644468	[91]	Subjects carrying the variant allele of rs4855094 or rs7644468 significantly enhanced the risk of gastroesophageal reflux disease to develop esophageal adenocarcinoma compared with subjects carrying homozygous wild genotypes
	IVS9+91	[92]	Mutation found in prostate tumours
	rs7651265	[83]	Association with rectal cancer
	C1241T; T1258C; del1352–1366; G1624A; G1633A; A1634G; C1636A; C1636G; A3140G; A3140T	[93]	Association with breast tumors and with significantly worse survival
	C3075T; gene amplification	[94]	These mutations have been found in thyroid cancer
	gene amplification; G1624A; G1633A; G353A; A331G	[95]	Mutations found in non-small-cell lung cancer
	rs2677760	[96]	This SNP was strongly associated with worse breast cancer disease-free survival in the overweight and obese patients
	G1624A; G1633A; A1928G; G3129A; A3140G	[97]	These SNPs have been found in bladder cancer
	G1633A	[98,99]	Mutation found in pancreatic neuroendocrine tumors and in squamous cell carcinoma
	Copy number variations	[100,101]	Amplification found in glioblastoma. Copy number variations found in diffuse large B-cell lymphoma had significantly shorter survival times
*PIK3CB*	Copy number variations	[101]	Association with significantly shorter survival times in diffuse large B-cell lymphoma
*PIK3C2B*	N232del; A577S; A577S	[64]	SNPs found in Müllerian adenosarcoma
*PIK3CD*	rs4129341	[102]	Association with a high risk to develop second primary tumors in patients with head and neck squamous cell carcinoma. The same variant genotype was also associated with significant benefit following 13-cis-Retinoic acid intervention
	Copy number variations	[100]	Found in Glioblastoma
*PIK3C2G*	T3130C	[62]	SNP found in intracranial germ cell tumours
*PRKCQ*	rs571715	[76]	Interactions between AKT3 rs12031994-PRKCQ rs571715 as well as AKT3 rs12031994-BID rs366542-PRKCQ rs571715 were significantly associated with disease aggressiveness in prostate cancer
*PIK3R1*	9-bp del	[100]	Mutation found in glioblastoma
	rs1862162	[68]	Association with risk of endometrial cancer and the hazard of death
	rs10515074	[73]	Association with survival in muscle invasive and metastatic bladder cancer patients
*PTEN*	rs701848	[61,81]	Association with the risk of recurrence and survival in gastric cancer patients and with an increased renal cell carcinoma risk
	G407A	[62]	SNP found in intracranial germ cell tumours
	rs12357281	[67]	Association with a decreased recurrence risk of esophageal cancer
	rs532678	[72]	This SNP, in association with PDK1 rs11904366 (G>T), PRKAG2 rs1881632 (C>T) and dietary fat and carbohydrate, influence the risk of both colon and rectal cancer
	gene loss	[85]	PTEN loss by itself or combined with mutated PIK3CA tended to confer radiosensitivity in breast cancer patients
	gene loss	[86]	PTEN protein expression was more often decreased or lost in endometrial carcinomas than colorectal cancer
	gene loss	[90]	Association with increased risk of death in the HER2-positive patients with metastatic breast cancer treated with Trastuzumab
	gene loss	[95]	PTEN loss was observed in non-small-cell lung cancer tumor samples with both squamous cell and adenocarcinoma histologies and render the cells sensitive to the PI3K inhibitor GDC-0941
	738delG; T323G; 961_962insTGACAAGGAATATCTAGTACTTACTTTAA; T202C; G494A	[98]	Mutations found in pancreatic neuroendocrine tumors
	R130X; L139X; R142Q; delAAGCT (codon 125-126); G165E; delAGAA (codon 183-184); delCCCT (codon 319-320)	[99]	Mutations found in squamous cell carcinoma and adenocarcinoma. Some are associated with loss of PTEN
	34-bp insertion in exon 7, a 4-bp deletion in exon 8, a 1-bp insertion in exon 7 and a point mutation in intron 3	[100]	Mutations found in glioblastoma
	rs1234221	[102]	Association with an high risk to develop second primary tumors in patients with head and neck squamous cell carcinoma and with significant benefit following 13-cis-Retinoic acid intervention
	gene loss	[103]	PTEN mRNA and protein levels were found to be significantly lower in medulloblastomas compared with normal cerebellar tissue of different developmental stages
	PTEN frame-shift deletion	[104]	Association with AKT hyper-activation in melanoma
	copy number variations	[105]	Association with lung tumorigenesis
	deletion	[106,107]	Association with early disease recurrence, reduced levels of androgen receptor expression and pAKT activation in prostate cancer
	deletion	[108]	Association with gastric carcinogenesis
*PTEN*	promoter polymorphisms (-903GA, -975GC, and -1026CA)	[109]	Association with worse long term survival and risk of distant metastasis in breast cancer patients
	rs701848; rs1903858	[110]	Association with decreased chronic obstructive pulmonary disease risk
	Deletion homozygosity (from D10S1765 to D10S541; from D10S215 to IVS4+109; from D10S215 to IVS8+32). Promoter region (1238A/G; 1110A/G; 1084C/T; 1000T/C; 930G/A; 920G/T; 895A/C; 861G/T; 834C/T; 764G/A)	[111]	Patients carrying the promoter mutations or deletions showed a decrease in PTEN protein of the correct molecular weight with nonfunctional lipid phosphatase activity and elevated level of phosphorylated Akt in patients with Cowden syndrome and patients with Bannayan-Riley-Ruvalcaba syndrome
	IVS1+41C>G; c.166T>G; c.70G>T; c.463T>A; 469–470insG; 741–742insA; c.862G>T; IVS3-1G>T; allelic loss	[112]	Association with reduced or absent PTEN protein expression in primary adenocarcinomas of the ovary
*RAPTOR*	rs9906827; rs7208502	[73]	Association with survival in metastatic bladder cancer patients
	rs11653499; rs7212142; rs7211818; rs7208536; rs4969444; rs2048753; rs2672890; rs9897968; rs1877926; rs2271612; rs6420481; rs1062935	[75]	Association with bladder cancer risk
	rs11653499, rs7211818, rs7212142; rs9674559	[113]	Association with bladder cancer risk
*RHEB*	rs717775	[75]	Association with bladder cancer risk
*RPS6KA5*	rs7155799	[75]	Association with bladder cancer risk
*RPS6KB1*	gained regions	[14]	This gene was highly amplified in estrogen receptor (ER)+/progesterone receptor (PR)− breast tumors compared with ER+PR+ tumors
*TSC1*	rs2519757	[96]	Association with improved disease-free survival in breast cancer
	rs7040593; rs3827665; rs739442; rs2519760; rs2809243; rs4962225; rs7035940; rs10491534; rs2073869; rs7874234; rs869116; rs4367688	[102]	Association with a high risk to develop second primary tumors in patients with head and neck squamous cell carcinoma. Rs739442, rs4962225 and rs7874234 were also associated with significant benefit following 13-cis-Retinoic acid intervention
	73-77Δ5; C104G; C163T; A203G; A314G; T473G; C555G; C585A; C616G; T648A; IVS7-1G>A; C866A; G1041A; C1250T; 1531insA; C1579T; 1727-1748Δ22insG; 1872ΔT; 1958-1959ΔTA; C2612G; IVS20+1G>A; C2851T	[97]	These mutations are common in bladder cancer
	rs13295634; rs11243940	[72]	*PDK1* rs11904366 (G>T) & *TSC1* rs11243940 (A>G) in combination with dietary fat and carbohydrate, influence the risk of both colon and rectal cancer.
	rs7874234	[83]	Association with a significant 40% reduction in the risk of rectal and colon cancer
	rs7874234	[114]	Association with age at diagnosis in ductal estrogen receptor (ER)+ breast carcinoma patients
*TSC2*	rs2073636	[75]	Association with bladder cancer risk
	p.A1429S; p.F1510del	[64]	Mutations found in Müllerian adenosarcoma
	rs3087631	[83]	Association with colon cancer
	C3422T; G4498A; 4113_4114delTG; C5383T; C26A; A4952G	[98]	These mutations are common in pancreatic neuroendocrine tumors
	rs13335638	[115]	Association with breast cancer
